# The Mitten

**Published:** 1864-03

**Authors:** 


### "THE MITTEN."

SEVENTEEN years ago there was a fair girl so pure, so lovely, so refined, that she still rises to my mind as almost akin to angels. She was wooed and ultimately won by a handsome young man of considerable wealth. He sported a fine team, delighted in hunting, and kept a fine pack of hounds. He neither played cards, drank wine, nor used tobacco. He had no occupation, no calling, no trade. He lived on his money, the interest of which alone would have supported a family handsomely. I never saw the fair bride again until a few days ago. Seventeen years had passed away, and with them her beauty and her youth; her husband's fortune and his life, during the latter part of which they lived in a log-cabin on the banks of the Ohio river, near Blennerhasset's Island; a whole family in one single room, subsisting on water, fat bacon, and corn bread. The husband had no business capacity. He was a gentleman of education, of refinement, of noble impulses; but when his money was gone he could get no employment, simply because he did not know how to do any thing. For a while he floundered about, first trying one thing, then another, but

"failure" was written on them all. He however finally obtained a situation; the labor was great, the compensation small; it was that or starvation; in his heroic efforts to discharge his duty acceptably he overworked himself and died, leaving his widow and six girls in utter destitution. In seventeen years the sweet and joyous and beautiful girl had become a broken-hearted, care-worn, poverty-stricken widow, with a houseful of helpless children!

Young woman! if a rich young man asks you to marry him, and has no occupation, or trade, or calling, by which he could make a living if he were thrown on his own resources, you may give him your respect, but "give him the mitten."

Whatever may be a young man's qualities, if he is fond, very fond of going to the theater, "refuse" him.

If a young man shows by his conversation that he is an admirer of fast horses, and is pretty well acquainted with the qualities and "time" of the best racing nags of the country, when he asks your hand, "give him the mitten" only.

If you ever hear a young man speak of his father or mother disrespectfully, contemptuously, do not encourage his attentions; he will do the same of you, and in many ways will make your heart ache before you die.

If you know a young man likes to stand around tavern-doors, at the street-corners, and about "groceries," cut your hand off rather than place it in his; he is worth only the "mitten."

If your suitor can tell you a great deal about cards; seems familiar with a multitude of "tricks" which can be performed with the same, and is himself an adept in such things, let him win all the money he may from others, but let him not "win" your heart, for he will "lose it" in a year, and leave you a broken one in its place.

If you know of a "nice young man" who will certainly heir a large estate, who is of a "highly respectable family," who seems to be at home as to the usages, customs, and proprieties of good society, and yet who is indifferent about attending church on the Sabbath-day, who speaks disparagingly of clergymen, who talks about religion in a patronizing way as "a very good thing in its place," particularly for old women, weak young girls and children, never marry him should he ask you.

Such a man can never warm a woman's heart; will never twine around it the tendrils of a true affection, for he is innately cold, unsympathizing and selfish, and should sickness and trouble come to you, he will leave you to bear them all alone.

Idleness, the having no occupation, will always and inevitably engender moral and physical disease; and these traits will be more or less perpetuated in the children born to such; the brunt of these calamities has to be borne by the mother, and in the bearing up against them, how many a noble-hearted woman has sorrowed, and grieved, and toiled herself into a premature grave, may never be known, but the number can not be expressed in a few figures. Therefore, my sunny-faced daughter, if you do not want to grow old before your time, to live a life of toil and sorrow, and then prematurely die, give not your hand, but only "the mitten" to a young man, however well born or rich, who has not a legitimate calling by which he could "make a living" if he were by some fortuity left penniless.

